# Use of near-infrared light to reduce symptoms associated with restless legs syndrome in a woman: a case report

**DOI:** 10.1186/1752-1947-4-286

**Published:** 2010-08-23

**Authors:** Ulrike H Mitchell

**Affiliations:** 1 Department of Exercise Sciences, Brigham Young University, Provo, Utah 84602, USA

## Abstract

**Introduction:**

We describe a potential new treatment option for patients suffering from restless legs syndrome. Contemporary treatment for restless legs syndrome consists mostly of dopaminergic drugs that leave some patients feeling nauseated and dizzy. A non-invasive, drug-free option would open new doors for patients suffering from restless legs syndrome.

**Case presentation:**

A 69-year-old Caucasian woman met International Restless Legs Syndrome Study Group criteria for the diagnosis of restless legs syndrome. She had been afflicted with restless legs syndrome for over 30 years and tried many of the available pharmaceutical remedies without success. For this study she received 30-minute treatment sessions with near-infrared light, three times a week for four weeks. The restless legs syndrome rating scale was used to track symptom changes; at baseline she scored "27" on the 0 to 40 point scale, which is considered to be "severe". Our patient was almost symptom free at week two, indicated by a score of "2" on the rating scale. By week four she was completely symptom free. The symptoms slowly returned during week three post treatment.

**Conclusions:**

The findings suggest that near-infrared light may be a feasible method for treating patients suffering from restless legs syndrome. Undesirable side-effects from medication are non-existent. This study might revive the neglected vascular mechanism theory behind restless legs syndrome and encourage further research into this area.

## Introduction

Restless legs syndrome (RLS) is a chronic sensorimotor disorder, characterized by a strong urge to move, accompanied or caused by uncomfortable or even distressing paresthesia of the legs, described as a creeping, tugging, "pulling" feeling. The symptoms often become worse throughout the day, leading to sleep disturbances or deprivation and, consequently, to impairment of alertness and daytime functions [1]. The symptoms are usually lessened by movement [2].

The diagnosis of RLS is clinical and based on a patient's description of the symptoms. In an attempt to standardize diagnostic procedures, the International Restless Legs Syndrome Study Group (IRLSSG) identified four criteria to substantiate the diagnosis of RLS [3]. To meet the criteria the patients had to answer the four questions affirmatively. The questions explore whether the subjects have an urge to move their legs, whether the symptoms begin or worsen during periods of inactivity, whether the urge to move is at least partially relieved by movement, and whether this urge to move is worse in the evening or night [3]. The IRLSSG also defined three supportive features. While they are not essential to the diagnosis of RLS, their presence can help resolve diagnostic uncertainty; they are: family history, presence of periodic limb movement and the response to dopaminergic treatment [3].

The IRLSSG developed the International Restless Legs Scale (IRLS) for measuring severity of the symptoms and their impact on a person's life [3]. The scale evaluates and reflects subjective assessment of the primary features, intensity, and frequency of the disorder and associated sleep problems as well as the impact of the symptoms on a patient's mood and daily functioning [4]. The 10-question scale has five response options with an associated score from "0" (no impact or symptoms) to "4" (severe), yielding a maximum score of 40. Hoegl and Gschliesser [5] reviewed several assessment tools used for RLS patients. They strongly support the use of the IRLS as the gold standard for assessing disorder severity. They also recommend it as a tool to follow changes in a subject's status and suitable for repeated measurements.

The pathophysiology of RLS is not fully clear. RLS can be classified into primary or secondary forms, delineating genetic and idiopathic contributions or involvement of other underlying pathologies respectively. Secondary RLS is usually dealt with by treating the underlying causes or associated medical conditions. For primary RLS dopaminergic medications are considered first line treatment for their effectiveness and usual rapid and dramatic improvement of the symptoms [6]. Other drugs, such as opioids (methadone, hydrocodone), GABA analogue (gabapentin, pregabalin), and benzodiazepines (clonazepam) are also used to treat moderate to severe RLS [6,7]. Until May 2005 there were no FDA-approved drugs on the market for the treatment of RLS. Now ropinirole and pramipexole, both dopamine agonists, are available. Unfortunately these drugs can cause insomnia, nausea, dyspepsia, and dizziness [8]. Since the drugs only provide symptomatic relief and are not considered a cure, the benefit of the treatment should justify any potential side effects and costs [6]. Non-pharmacological treatment of RLS includes improving sleep quality by controlling sleep times, reducing caffeine and alcohol consumption, and maintaining a daily moderate exercise program [9]. The efficacy of these options has not been well documented and is limited.

Promising alternative treatment choices are welcomed options. One of them might already be on the market, but is currently used for other disorders: near-infrared light (NIR). It is utilized for patients with neuropathy to increase sensation and decrease pain. NIR has a wavelength of 880 nm to 890 nm and is emitted through diodes [10]. For this case report Anodyne was used, but there are other similar devices available for healthcare providers. Anodyne is FDA approved for increasing circulation and reducing pain, and it has been successfully used in wound management [11]. Researchers hypothesize that the success of NIR treatment lies in its ability to increase bioavailability of nitric oxide (NO) in the lumen. In 1992 NO was hailed as the molecule of the year for its significant role in vasoregulation, neurotransmission, signal transduction, anti-microbial defense, and digestion [12]. It is produced by the enzyme nitric oxide synthase (NOS-3), which is activated by, among other factors, shearing forces generated by blood flow that act on the vascular endothelium [13]. Nitric oxide is also found tightly bound to the hemoglobin contained in erythrocytes. It has been suggested that NO can be released from this bond through intensive illumination [14]. Once generated, NO initiates a cascade of events, leading to vasodilation and increased blood flow.

After being treated for neuropathy for 30 minutes with NIR, three times a week for four weeks, three patients reported that, while their neuropathy was better, they were more excited that their RLS symptoms had either decreased or been eliminated. These findings prompted this investigation into the effectiveness of NIR therapy for the treatment of symptoms associated with RLS.

## Rationale

Treatment with NIR has been shown to increase blood flow, possibly due to its ability to generate NO in the endothelium. Nitric oxide has also been linked to improved neurotransmission. It is thus conceivable that tissue treated with NIR could impact RLS, a neurological disease, and decrease the symptoms associated with it.

This case report was part of a randomized, controlled study (not yet published), which was approved by the institutional review board at Brigham Young University, Provo, Utah.

The purpose of this report is, therefore, to describe an investigation that was conducted on the effectiveness of using NIR to decrease symptoms associated with RLS. Written informed consent was obtained from the patient for publication of this case report. A copy of the written consent is available for review by the Editor-in-Chief of this journal.

## Case presentation

A respondent to a newspaper advertisement with symptoms of RLS, was recruited for this case report. During the evaluation she was asked about her symptoms--RLS can only be diagnosed based on subjective findings--and she met the four IRLSSG criteria [3].

Our patient was a 69-year-old Caucasian woman (1.63 m, 63.5 kg) who described her general health status as "good". Her activity level was "reasonably active"; she walked in the mornings and did some occasional yoga. She did not complain of any mobility decreases and enjoyed good flexibility. Her sleep pattern was disturbed, mostly because of her RLS symptoms. Her urge to move her legs was especially strong every evening. She had difficulty falling asleep and could only do so after taking zolpidem 10 mg (Ambien). She also reported having been diagnosed with depression and had taken 20 mg fluoxetine (Prozac) daily for almost 25 years. Our patient never made a connection or noticed a correlation between the antidepressant and RLS symptoms. She complained of constant tiredness and fatigue, due to restless sleep. Our patient was not aware of any other family member before her suffering from RLS. Her father had "circulation problems" in his legs, but details are unknown. However, both of her daughters, aged 43 and 39, reported symptoms of RLS. Neither of them was taking medication for RLS. Our patient's chief complaints were painful sensations in her legs and hips, triggering an urge to move the legs, as well as sleep disturbance. Her social life suffered due to her inability to sit still when going to the movies or the theater or when flying in a plane. She remembered having suffered from RLS before she knew her symptoms had a name--that was about 30 years ago. Since then the symptoms had become more pronounced. For many years she did not receive treatment for the symptoms, because doctors did not recognize her condition--until four years ago, when her family doctor diagnosed her with RLS. At that time she was given muscle relaxants (names not known), but they did not change her symptoms. Consequently, she was given a benzodiazepine (Clonazepam) combined with a sedative (zolpidem). Although her sleep improved, the symptoms associated with RLS remained. When ropinirole became available on the market as one of two FDA approved drugs for RLS, she tried the Starter Kit, where the pills with increasing strength were marked each day they needed to be taken. After less than two weeks she discontinued taking the drug because it made her feel "horrible". Our patient does not remember having had a positive response from the drug, just side effects. The side effects included nausea, balance problems, impaired thinking ability, and, worst of all, remaining RLS symptoms. Our patient was not aware of ever having periodic limb movements, in sleep or at rest.

She responded to the newspaper advertisement for this study because she hoped that some treatment would be available for her. She gave written informed consent to take part in this trial.

Vital signs: blood pressure is 120/78 with a pulse rate of 68. Sensation in lower extremities including feet was intact as measured with Semmes-Weinstein monofilament. The patient was non-diabetic.

Pathologies such as hypertension, arthritis, gastroesophageal reflux disease, depression, anxiety, and diabetes, as well as several lifestyle factors such as increased body mass index, lower income and being unemployed, smoking, lack of exercise, less than six hours of sleep, and low alcohol consumption are linked to this disorder [15]. With the exception of depression, our patient had none of the above.

Our patient exhibited normal range of motion in upper and lower extremities and trunk. Strength was graded 5/5 in all major muscle groups.

The history, systems review, and other examination findings seemed to corroborate her diagnosis of RLS; the differential diagnosis of neuropathy could be excluded.

Based on anecdotal evidence of NIR reducing symptoms associated with RLS, our patient received twelve 30-minute NIR treatment sessions. This is the same protocol that is used nationwide for neuropathy treatment.

The treatments were administered three times a week for four weeks. No other treatment was given, and our patient was asked not to change anything in her daily routine. She lay comfortably on a treatment bench in a quiet room at 21°C (+1°). For comfort, the knees were supported by a five-inch bolster. The lower leg skin area was covered with plastic wrap, which acted as a barrier between skin and diodes to ensure compliance with infection control procedures. Eight flexible monochromatic near-infrared photo energy diodes (60 on each pad) were placed on the lower legs. During each treatment the output was adjusted to the highest level of intensity. After a 30-minute supervised treatment period with NIR, the diodes and plastic wrap were removed. During the Anodyne treatment our patient received an 890 nm wavelength light, pulsed at 292 times/s, with a power output of 600 mW/cm^2^. Our patient was asked to fill out a validated RLS self-rating scale[4] in the week before treatment, at the end of each treatment week, one week after and three weeks after cessation of treatment. It was determined that treatment with NIR therapy was deemed to be successful if the patient improved by 10 points on the scale after four weeks of treatment.

**Figure 1:**
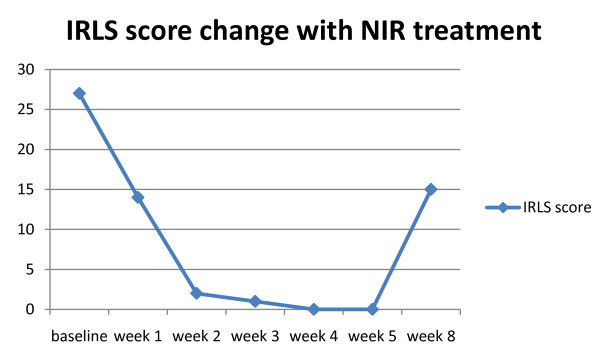
**Patient's IRLS scores indicate resolution of RLS symptoms**.

Our patient scored a "27" (out of "40") at her first visit, "14" after her first treatment week, "2" after her second week, and "1" after her third week. Weeks four and five were scored a "0" (no symptoms) (Figure 1). The symptoms associated with RLS decreased from "severe" (27/40 possible points on IRLS) to "no symptoms" (0/40 possible points on IRLS) after four weeks of treatment. Our patient stated that she felt marked improvement in every aspect of living. In her own words, --It has changed my "life". Our patient reported that the symptoms returned slowly during week seven and were at a "15" by the end of week eight (four weeks post treatment).

## Discussion

The pathophysiology of RLS is not clear. In the 1940s and 1950s it was hypothesized that decreased blood flow was responsible for the symptoms associated with RLS [16]. Ekbom [2] believed that vasodilators given to RLS sufferers would decrease the symptoms. Today it is widely accepted that the central nervous system is involved in RLS, but the original hypothesis of a vascular association still exists. One study reports that increased vascular blood flow with enhanced external counter pulsation significantly decreased RLS symptoms in six patients [16]. Another study [17] showed a high prevalence (36%) of RLS in patients presenting with chronic venous disorder. The author of this case report theorizes that the symptoms associated with RLS could stem from a feedback mechanism where decreased tissue perfusion in the legs signals to the brain the need to move. Activity, such as movement or walking, increases blood flow to the muscle and tissue [18]. The proposed mechanism of NIR therapy is its ability to generate NO in the endothelium [19] and even in the lumen directly by dissociating NO from hemoglobin contained in erythrocytes [14,20]. Nitric oxide is able to initiate and sustain vasodilation [21,22] and, as a neurotransmitter itself, has influence on neurotransmission [22]. Phototherapy, which includes NIR, has been known to decrease pain by changing cell membrane permeability. This leads to enhanced synthesis of endorphins, increased nerve cell potential and hence to pain relief [23]. NIR consequently can affect three factors associated with RLS: vasodilation [16], neurotransmission [24] and pain relief [25]. It is thus conceivable that NIR could positively impact this pathology. Recent findings could validate this hypothesis as well as function as the missing link between theory and fact. A German study [26] discovered significant evidence for an association of RLS with sequence variations in the NOS1 gene, pointing to a possible involvement of the NO/arginine pathway in RLS disease susceptibility and in the etiology of RLS.

Other factors may have contributed to our patient's improvement. As in the study by Ferini-Strambi *et al*. [7], where IRLS scores decreased in medicated and non-medicated RLS patients after taking part in weekly group sessions, the social interaction between therapist and subject could have contributed to her improvement. However, the therapist/subject interaction in this case report was kept within the limits of a typical therapist/patient relationship and was not intended or designed to have a "support group" character.

A recent meta-analysis [27] assessing the placebo effect in RLS treatment studies found a substantial placebo response associated with RLS treatment. This response was greater for the IRLS compared to other scales, possibly related to its multidimensional assessment character. On average, more than one-third of RLS subjects experienced a major improvement of RLS symptoms while receiving placebo treatment. The author proposes that the reason for this might be related to the unique responsiveness of RLS to dopaminergic agents and opioids - both systems implicated in the placebo response. The question of whether our patient's improvement was likely due to a pure placebo effect can only be answered by conducting a randomized controlled trial.

## Conclusions

This case report shows how NIR helped one patient suffering from RLS symptoms to eliminate her symptoms and suggests that this protocol might be a potential treatment option for other, similar patients. One patient received 30-minute NIR treatment sessions, three times a week for four weeks. This regimen was taken from a protocol used in home health to treat patients with neuropathy. If treatment with NIR could be used to alleviate RLS symptoms, the patients would be able to benefit greatly from this non-invasive option.

This report adds to existing studies as it suggests a different, non-drug-related treatment option to patients who would otherwise have to take dopaminergic or other drugs. The mechanisms with which NIR can alleviate RLS symptoms are not clear. One supposition can be made: light has been shown to generate NO in the endothelium, which through a cascade of events leads to vasodilation. Vasodilation is also the result of exercise [18], one of the few non-drug related treatment options that decreases RLS symptoms. While no direct relationship between NO and RLS symptoms can be shown, it is plausible that this radical, generated in the lumen of blood vessels, might have similar benefits to the patients as exercise. Further research into this hypothesis is suggested.

It is of course too early to suggest that treatment with NIR is the best treatment option for patients suffering from RLS; a randomized clinical trial would shed more light on the usefulness of this treatment.

## Consent

Written informed consent was obtained from the patient for publication of this case report and any accompanying images. A copy of the written consent is available for review by the Editor-in-Chief of this journal.

## Competing interests

The author declares that they have no competing interests.
